# PopHumanVar: an interactive application for the functional characterization and prioritization of adaptive genomic variants in humans

**DOI:** 10.1093/nar/gkab925

**Published:** 2021-10-19

**Authors:** Aina Colomer-Vilaplana, Jesús Murga-Moreno, Aleix Canalda-Baltrons, Clara Inserte, Daniel Soto, Marta Coronado-Zamora, Antonio Barbadilla, Sònia Casillas

**Affiliations:** Department of Genetics and Microbiology, Universitat Autònoma de Barcelona, Bellaterra, Barcelona 08193, Spain; Department of Genetics and Microbiology, Universitat Autònoma de Barcelona, Bellaterra, Barcelona 08193, Spain; Institute of Biotechnology and Biomedicine, Universitat Autònoma de Barcelona, Bellaterra, Barcelona 08193, Spain; Department of Genetics and Microbiology, Universitat Autònoma de Barcelona, Bellaterra, Barcelona 08193, Spain; Institute of Biotechnology and Biomedicine, Universitat Autònoma de Barcelona, Bellaterra, Barcelona 08193, Spain; Department of Genetics and Microbiology, Universitat Autònoma de Barcelona, Bellaterra, Barcelona 08193, Spain; Department of Genetics and Microbiology, Universitat Autònoma de Barcelona, Bellaterra, Barcelona 08193, Spain; Institute of Biotechnology and Biomedicine, Universitat Autònoma de Barcelona, Bellaterra, Barcelona 08193, Spain; Department of Genetics and Microbiology, Universitat Autònoma de Barcelona, Bellaterra, Barcelona 08193, Spain; Institute of Biotechnology and Biomedicine, Universitat Autònoma de Barcelona, Bellaterra, Barcelona 08193, Spain; Department of Genetics and Microbiology, Universitat Autònoma de Barcelona, Bellaterra, Barcelona 08193, Spain; Institute of Biotechnology and Biomedicine, Universitat Autònoma de Barcelona, Bellaterra, Barcelona 08193, Spain

## Abstract

Adaptive challenges that humans faced as they expanded across the globe left specific molecular footprints that can be decoded in our today's genomes. Different sets of metrics are used to identify genomic regions that have undergone selection. However, there are fewer methods capable of pinpointing the allele ultimately responsible for this selection. Here, we present PopHumanVar, an interactive online application that is designed to facilitate the exploration and thorough analysis of candidate genomic regions by integrating both functional and population genomics data currently available. PopHumanVar generates useful summary reports of prioritized variants that are putatively causal of recent selective sweeps. It compiles data and graphically represents different layers of information, including natural selection statistics, as well as functional annotations and genealogical estimations of variant age, for biallelic single nucleotide variants (SNVs) of the 1000 Genomes Project phase 3. Specifically, PopHumanVar amasses SNV-based information from GEVA, SnpEFF, GWAS Catalog, ClinVar, RegulomeDB and DisGeNET databases, as well as accurate estimations of iHS, nS_L_ and iSAFE statistics. Notably, PopHumanVar can successfully identify known causal variants of frequently reported candidate selection regions, including *EDAR* in East-Asians, *ACKR1* (*DARC*) in Africans and *LCT/MCM6* in Europeans. PopHumanVar is open and freely available at https://pophumanvar.uab.cat.

## Graphical Abstract

**Graphical Abstract:**
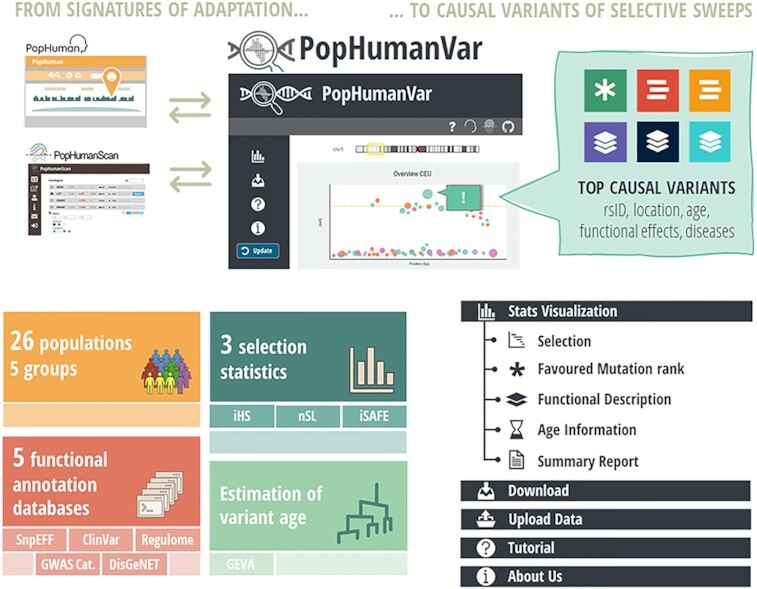
PopHumanVar is an interactive online application that is designed to facilitate the exploration and thorough analysis of candidate genomic regions by integrating both functional and population genomics data currently available. PopHumanVar generates useful summary reports of prioritized variants that are putatively causal of recent selective sweeps.

## INTRODUCTION

The landscape of variation in human genomes holds the record of our evolutionary history. Despite the numerous attempts to identify selection targets in diverse populations (1–5), or date the time of appearance of an adaptive mutation and trace its spread around the globe (6–11), how, where, and when our genomes underwent adaptation is a subtle issue which is far from being resolved.

One of the results of next-generation sequencing (NGS) technologies is the 1000 Genomes Project (1000GP) (12), an international research effort to generate a catalog of human genetic variation. Years after its completion, it still represents one of the largest public catalogs of human variation and genotype data. Reporting >84 million variants, with 2504 sequenced genomes from 26 populations, it is one of the main references for population genomics in the human species.

With the 1000GP, came the possibility of scanning the entire genome for signatures of natural selection, resulting in the piling up of genomic regions believed to have evolved under positive selection (13–18). However, these genome-wide scans present two major constraints: the scarce agreement among studies, and the lack of in-depth characterization of candidate loci. In 2018, we tackled the first constraint by presenting PopHumanScan (19), a genome-wide catalog that brings together 2859 candidate regions under selection resulting from the combination of several metrics that capture selection in a wide range of time scales and selective regimes. Even though PopHumanScan compiled an exhaustive list of candidate regions and cross-referenced them to 268 previous publications, it did not provide tools to facilitate their validation nor to perform thorough analyses at the single nucleotide variant (SNV) level.

Integrating the numerous currently available information layers on functional and population genetics metrics can help portray the genomic landscape of a putatively selected region and aid the prioritization of causal genetic variants. These sources range from functional annotations (e.g. associations with phenotypes and diseases, implication in the regulation of gene expression, or predicted functional effects), to selection statistics based on the analysis of genomics data, to genealogical estimations of variant age. As far as we know, even though several SNV-oriented public online databases exist that cover one of the previous aspects (see e.g. snpXplorer (20)), none of them bring both functional and evolutionary information all together with the main focus of identifying causal variants of selective sweeps.

Here, we present PopHumanVar, an interactive online application that is designed to facilitate the exploration and thorough analysis of candidate genomic regions under selection, generating useful summary reports of prioritized variants that are putatively causal of recent selective sweeps. It compiles and graphically represents selection statistics based on linkage disequilibrium, a comprehensive set of functional annotations, and recent genealogical estimations of variant age for SNVs of the 26 populations of the phase 3 of the 1000GP. Specifically, PopHumanVar gathers data either computed or compiled from the following data sources: the Integrated Haplotype Score (iHS) (21), the Number of Segregating sites by Length (nS_L_) (22), the Integrated Selection of Allele Favored by Evolution (iSAFE) (23), SnpEFF (24), RegulomeDB (25), ClinVar (26), GWAS Catalog (27), DisGeNET (28), and the Genealogical Estimation of Variant Age (GEVA) as obtained from the Human Genome Dating database (or Atlas of Variant Age) (29). As such, PopHumanVar is complementary to our previous genome browser -PopHuman (30)- and database of candidate selection regions -PopHumanScan (19)-, allowing researchers to focus on particular selective sweeps, pinpoint the corresponding causal variants, and estimate variant age. For populations and/or samples not included in the online application, PopHumanVar allows uploading and analyzing a VCF file with custom data.

The utility of PopHumanVar has been tested on frequently reported candidate genomic regions in genome-wide scans for positive selection in humans, including a region close to the gene *EDAR*, which is associated with hair follicle thickness and straightness and shovel-shaped incisors in East-Asians (31–34), a region in the gene *ACKR1* (*DARC*), which is associated with resistance to malaria in Africans (35–37), as well as a region close to the genes *LCT* and *MCM6*, which is associated with lactase persistence in Europeans (38,39). In all three cases, PopHumanVar is able to identify the causal variant reported in previous studies and accurately estimate the variant age. These promising results illustrate the exploratory potential of PopHumanVar to push out into yet unfamiliar human adaptation signatures, including those compiled in PopHumanScan or the ones that can be visually extracted from PopHuman, but also any other genomic region of interest.

## CONTENTS OF POPHUMANVAR

PopHumanVar collects evolutionary data, functional annotations, and age information altogether. Evolutionary and age information have been computed on the 26 populations of the phase 3 of the 1000GP (12), while functional annotations have been retrieved from publicly available databases (see below). In total, 81.70 M SNVs of the 1000GP have information for one or more of the collected data sources.

### Selection statistics and favored mutation rank

All selection statistics were computed on the 26 populations of the phase 3 of the 1000GP, including non-inbred individuals as specified by Gazal *et al.* (40). We considered autosomal biallelic SNVs that are accessible to sequencing techniques according to the 1000GP pilot accessibility mask (12). We advise taking results for the four admixed-American populations with caution, as these populations have complex recent demographic histories that may mimic some patterns of genetic diversity that PopHumanVar uses to infer selection, and thus results from these populations may be difficult to interpret.

#### Integrated Haplotype Score (iHS)

**Figure 1.**
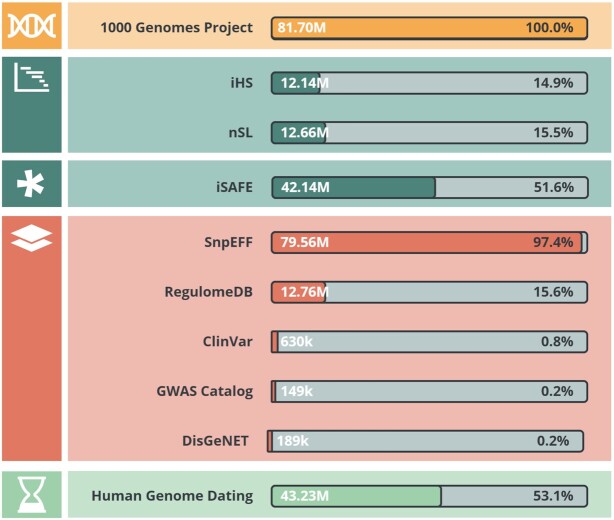
Summary of the contents of PopHumanVar. Bars represent the number and percentage of single nucleotide variants (SNVs) with information for each dataset.

Defined by Voight *et al.* (21), it tracks the decay of haplotype homozygosity for both ancestral and derived haplotypes. It has good power to detect selective sweeps at a moderate frequency (50–80%) (16,21,41). iHS was computed with selscan v1.2.0a and norm v1.2.1a (42). We only considered those SNVs having a Minor Allele Frequency (MAF) higher than 0.05 and a maximum gap of 20 kb between consecutive SNPs when assembling haplotypes. The recombination maps used to interpolate genetic positions (necessary to compute iHS) were the sex-averaged ones from Bhérer et al. (43). We obtained estimates for a total of 12.14 M SNVs (Figure 1). Significance was assessed from the empirical distribution of iHS values in each population separately.

#### Number of Segregating sites by Length (nS_L_)

It is also a haplotype-based statistic. It combines information on the distribution of fragment lengths, defined by pairwise differences, with the distribution of the number of segregating sites between all pairs of chromosomes (22). It is better than iHS at capturing soft sweeps. nS_L_ was also computed with selscan v1.2.0a and norm v1.2.1a. We only considered those SNVs having a MAF higher than 0.05 and a maximum gap of 20 kbp between consecutive SNPs when assembling haplotypes. We obtained estimates for a total of 12.66 M SNVs (Figure 1). As for iHS, significance was assessed from the empirical distribution of nS_L_ values in each population separately.

#### Integrated Selection of Allele Favored by Evolution (iSAFE)

It aims to identify the specific variant ultimately responsible for a selective sweep (23). iSAFE exploits coalescent-based signals in the surroundings of a candidate region to rank mutations according to their likelihood of having caused the selective sweep. In order to compute iSAFE genome-wide, we analyzed overlapping sliding windows of 3 Mbp, with a 1 Mbp overlap, all along the autosomal chromosomes (values suggested by the iSAFE authors in their GitHub repository at https://github.com/alek0991/iSAFE). From each window, we kept values for the 1 Mbp middle chunk and discarded values in the shoulders. In order to facilitate the genome-wide approach, we ran iSAFE with default parameters, but ignoring the gaps and increasing the maximum rank parameter up to the window size (MaxRank = window = 300) in order to retrieve values for all SNVs in the window. We obtained iSAFE values for a total of 42.14 M SNVs (Figure 1). Significance was assessed from the empirical distribution of iSAFE values in each population separately.

### Functional annotations

#### SnpEFF

It predicts and annotates the functional effects of genetic variants (24) (e.g. stop gain, splice donor variant, missense variant, intergenic region…), which are classified into four different categories based on their impact (i.e. high, moderate, low or modifier). We ran SnpEFF v.5.0 with default parameters and obtained annotations for 79.56 M SNVs (Figure 1). Affected genes, if any, were also recorded.

#### RegulomeDB

It predicts and annotates the regulatory potential of intergenic variants (25). Evidence is compiled from GEO (44), ENCODE (45), and the published literature, and it includes known, as well as predicted, regulatory DNA elements, such as regions of DNase hypersensitivity sites, transcription factor binding sites, and promoter regions that have been biochemically characterized to regulate transcription. RegulomeDB scores the regulatory potential of intergenic SNVs based on overlapping supporting information (i.e. 15 different scores, from 1a to 7). We retrieved RegulomeDB v.2.0.3 scores for 12.76 M SNVs (Figure 1).

#### ClinVar

It is one of the largest catalogs of genetic variants that are clinically associated with diseases, together with supporting evidence (26). It rates variant-disease associations into different categories (e.g. pathogenic, risk factor, presenting drug response, protective, benign…). We retrieved ClinVar (updated on 2021/03/04) annotations for 630 k SNVs (Figure 1).

#### GWAS Catalog

It is a quality-controlled, manually-curated, literature-derived collection of all published genome-wide association studies (GWAS) assaying at least 100 000 genetic variants (27). We retrieved the number of associations in the GWAS Catalog v1.0.2, as well as the specific traits reported, over 149 k SNVs (Figure 1).

#### DisGeNET

It is one of the largest publicly available collections of genes and variants associated with human diseases (28). It integrates data from expert-curated repositories, homogeneously annotated with controlled vocabularies and community-driven ontologies. It provides original metrics to assist the prioritization of genotype-phenotype relationships, such as disease specificity, evidence index, or number of Pubmed identifiers. We retrieved DisGeNET v.7.0 annotations for 189 k SNVs (Figure 1).

### Age estimation

#### Human Genome Dating (or Atlas of Variant Age)

It gathers age estimation results for more than 45 M variants in the human genome, computed using the Genealogical Estimation of Variant Age (GEVA) (29). GEVA is a method that exploits coalescent modeling to infer the time to the most recent common ancestor (TMRCA) between individual genomes based on three different clock models, considering: (i) mutation events that occur independently in each lineage and pile up as the ancestral haplotype is passed on over the generations (i.e. mutational clock); (ii) recombination events that shorten the length of the ancestral haplotype, independently in each lineage and across generations (i.e. recombination clock); or (iii) both (i.e. joint clock). We retrieved age estimates, as well as the corresponding quality scores, for all three clock models, from the Atlas of Variant Age database (downloaded on 2021/06/26) for a total of 43.23 M SNVs (Figure 1).

## OVERVIEW OF THE POPHUMANVAR INTERFACE

**Figure 2.**
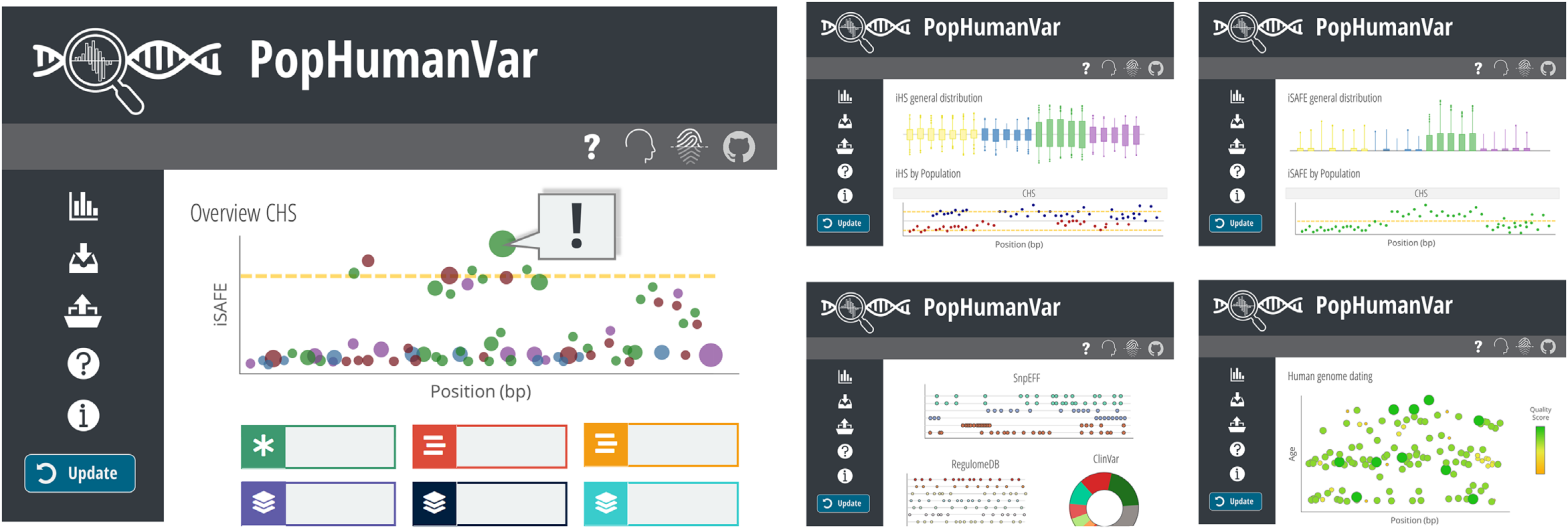
Simplified representation of the PopHumanVar interface. Some representative graphs of each of the five elements of the *Stats Visualization* section of the database are represented; from left to right, top to bottom: *Summary Report*, *Selection*, *Favored Mutation*, *Functional Description* and *Age Information*.

The PopHumanVar interface is divided into four main sections: (i) *Stats Visualization* represents the main navigation interface and provides several interactive graphs to aid the exploration and prioritization of genomic variants in the region of interest (Figure 2); (ii) *Download* provides tools to customize batch downloads from the database; (iii) *Upload Data* allows uploading and analyzing a VCF file with custom data; and (iv) *Tutorial* describes the database and presents a step-by-step usage example.

### Stats visualization

*Stats visualization* unfolds five subitems, each pointing to a visualization tab with one or more interactive graphs and tables. Note that while in the *Stats Visualization*, an additional menu –FILTERS MENU– adds to the left-side panel of the application. It allows: (i) choosing a genomic region of interest, either by entering its coordinates (GRCh37/hg19) or by searching a variant rsID, gene symbol or Ensembl identifier; (ii) activating one or more populations for which to display selection statistics and favored mutation ranks; and (iii) setting filters and parameters specific to the different visualization tabs. Changes in the FILTERS MENU will only be applied after clicking the ‘Update’ button at the bottom of the panel.

#### Selection (iHS & nS_L_)

iHS and nS_L_ value distributions within and across the genomic region of interest are displayed for each of the selected populations. Significant values are indicated by the golden horizontal lines and in the interactive hover panel, and can be filtered from the FILTERS MENU (default empirical *P*-value ≤ 0.005, customizable by the user).

#### Favored mutation (iSAFE)

As above, iSAFE value distributions within and across the genomic region of interest are displayed for each of the selected populations. For simplicity, only scores higher than 0.05 are represented. Significant values are indicated by the golden horizontal lines and in the interactive hover panel, and can be filtered from the FILTERS MENU (default empirical *P*-value ≤ 0.0001 following Akbari *et al.* (23), customizable by the user).

#### Functional description

It includes different representations of the annotations retrieved or computed from SnpEFF, RegulomeDB, ClinVar, GWAS Catalog and DisGeNET. Several filters can be applied from the FILTERS MENU.

#### Age information

Genealogical estimations of variant age are represented for each SNV across the genomic region of interest. Several filters and parameters can be applied from the FILTERS MENU, including the clock model and the units of age in generations or years. In the graph, size and color represent the quality score of the estimations.

#### Summary report

This section aims to summarize all the evolutionary and functional information gathered for the genomic region of interest through a JBrowse implementation showing gene annotations overlapping the region, direct links to PopHuman (30) and PopHumanScan (19), an eloquent summary graph including selection statistics and functional data, a list of top-20 automatically-prioritized putatively causal variants, and representations of EHH and haplotype furcations around any SNV of the top-20 prioritized variants (by default the one having the most extreme iSAFE value; computed with rehh (46)). In the summary graph, iSAFE scores are represented for all SNVs across the genomic region of interest. Color represents the strongest SnpEFF functional effect of each variant, and size represents its combined iHS + nS_L_ value. All the information displayed in this section refers to a specific population, which can be chosen from the right-side menu –DISPLAY OPTIONS–. Changes in the DISPLAY OPTIONS menu will be applied after clicking the ‘Refresh’ button at the bottom of the panel.

### Download

*Download* unfolds two subitems: *Current Region* and *Batch Download*. Both are used to download PopHumanVar data in tabular files. *Current Region* is the most customizable option and allows specifying how filters should be applied and which data should be included in the downloaded files. The right-side menu is used to set these parameters. *Batch Download* is used to make bulk downloads of the whole database contents. Data for a maximum of 50 Mbp (which may be split into several regions) can be retrieved at a time. Alternatively, data for whole chromosomes can be downloaded in compressed tabular files.

### Upload data

This section allows uploading a VCF file with custom data, which may cover up to 2 Mbp of genomic sequence for one single population. The data is processed automatically by the PopHumanVar pipeline and results are sent by email as a dynamic Shiny markdown file.

### Tutorial

This section documents the data used and the procedures implemented in PopHumanVar, and includes a complete tutorial introducing the usage of the database through a step-by-step worked example.

## POPHUMANVAR WITH AN EXAMPLE: SELECTION AT THE *EDAR* LOCUS

Here, we illustrate the usage of PopHumanVar with an example. This section summarizes the main findings, while the *Tutorial* section of the application contains a step-by-step guide of the same example.

For this case study, we will focus on a genomic region of 1.15 Mbp in chromosome 2 (chr2:109500927–109615828; GRCh37/hg19). The region contains the gene *EDAR* –*Ectodysplasin A Receptor*–, a cell-surface receptor that, upon binding to its ligand, induces an intracellular cascade leading to the activation of the transcription factor NF-κB.

**Figure 3.**
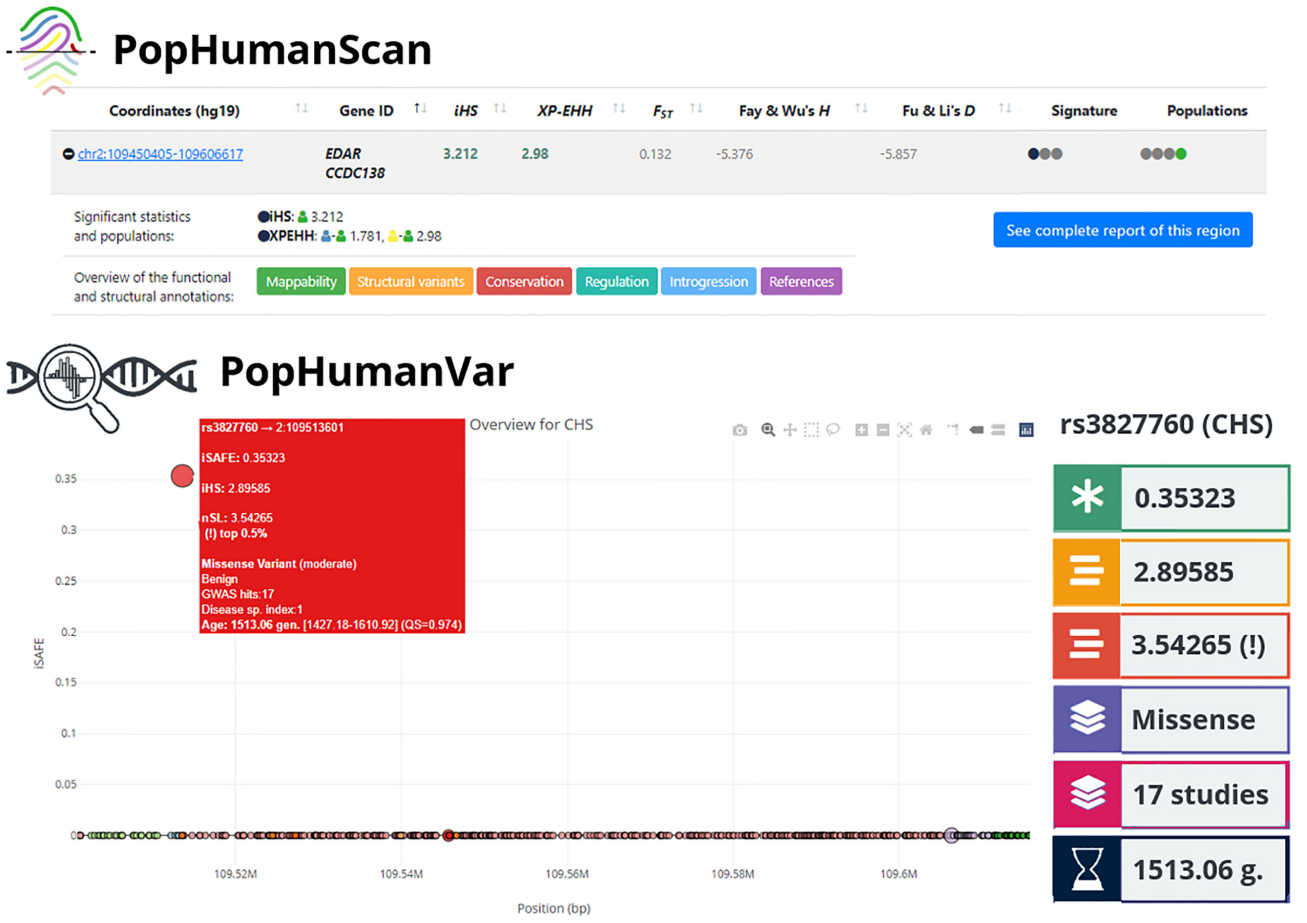
Characterization and variant prioritization in the *EDAR* gene region, which is associated with hair follicle thickness and straightness and shovel-shaped incisors in East-Asians. Complementary information obtained from PopHumanScan (top) and PopHumanVar (bottom) is shown. Color labels at the right of the PopHumanVar section represent, from top to bottom: *iSAFE*, *iHS*, *nS_L_* (top 0.5%), *SnpEff* effect, *GWAS Catalog* hits, and *Atlas of Variant Age* variant age in generations.

*EDAR* is a well-studied gene involved in the development of hair follicles, teeth, and sweat glands (31–34). It has frequently been reported in genome-wide scans for positive selection in humans (19,47–49) and is one of the candidate regions cataloged in PopHumanScan (Figure 3). PopHumanScan reports signatures of selection for haplotype-based statistics (i.e. iHS and XP-EHH) in East-Asian populations, especially in the Southern Han Chinese (CHS) population. In addition, the region shows extreme values (i.e. more than two standard deviations away from the mean value) both for the haplotype-based statistics iHS and XP-EHH, and the Site Frequency Spectrum (SFS)-based statistics Tajima's D and Fay and Wu's H, as displayed in PopHuman. Although both PopHuman and PopHumanScan bring our attention to this region, none of them allows us to shift to the SNV level and determine which variant was selected and when.

Instead of reporting summary statistics for a genomic region of interest, as PopHuman and PopHumanScan do, the PopHumanVar application presented here reports information at the SNV level and helps prioritize causal variants of selective sweeps. In the *EDAR* gene region, apart from gathering abundant functional annotations and evolutionary statistics, PopHumanVar prioritizes the protein-coding missense variant rs3827760 (A > G) as the top causal variant (Figure 3), which happens to be the known causal variant of a well-studied selective sweep in East-Asians (34,49). The derived G allele (Val370Ala substitution) is found at high frequency in East-Asian populations (87%), as well as Native American populations (39%) (31). It was driven to high frequency in East-Asia by positive selection prior to 10 000 years ago (6,31). In the GWAS catalog, it is reported to be associated with ear, eyebrow and chin morphology, and male-pattern baldness. The prioritized variant rs3827760 reports the highest iSAFE—as well as iHS and nS_L_—values in the region.

Two additional case studies, that of genes *ACKR1* (*DARC*) in Africans and *LCT/MCM6* in Europeans, are shown in the Supplementary Data.

## CONCLUSION

The PopHumanVar interactive application presented here, successfully tested by confirmatory results on the *EDAR* gene and two other well-known case studies, demonstrates its exploratory potential to prioritize variants in regions holding signatures of natural selection. Contrary to other SNV-oriented public online databases, the PopHumanVar approach brings both functional and evolutionary information all together, including natural selection statistics, functional annotations and genealogical estimations of variant age, and goes one step forward in the task of identifying and dating the emergence of variants that were putatively causal of the corresponding selective sweeps. In this way, PopHumanVar eases the description and thorough analysis of yet unfamiliar human adaptation signatures such as those compiled in PopHumanScan or the ones that can be visually extracted from PopHuman. Future implementations to PopHumanVar will include the development of a pre-processing module that returns uniform, adequate data from any human variation source data, so that additional populations not in the 1000GP, or new 1000GP samples, can be easily incorporated in PopHumanVar. All in all, we think that the public release of PopHumanVar will help advance our understanding of how environmental and social challenges have shaped our genomes through the action of natural selection.

## IMPLEMENTATION AND AVAILABILITY

PopHumanVar is based on the Shiny framework (50) for development of web-based applications using the R programming environment (51). Interactive plots are implemented with plotly (52). Interactive tables are generated with DT (53), an R-based interface to the JavaScript DataTables library. The genome browser integrated into the *Summary Report* section is implemented using the JBrowseR package (54). User queries are processed by R and sent to a MariaDB database. All scripts are available in the GitHub repository (https://github.com/ainacolovila/PopHumanVar).

PopHumanVar is served with Apache on a CentOS 7.2 Linux x64 server with 16 Intel Xeon 2.4 GHz processors and 32 GB RAM. All data, tools and support resources provided by the PopHumanVar database are open and freely available at https://pophumanvar.uab.cat. PopHumanVar is accessible and legible on computer, phone and tablet screens.

## Supplementary Material

gkab925_Supplemental_File

## Notes

Present address: Marta Coronado-Zamora, Institute of Evolutionary Biology, CSIC-Universitat Pompeu Fabra, Barcelona 08003, Spain.
