# Supplementary material for: PopHumanVar: an interactive application for the functional characterization and prioritization of adaptive genomic variants in humans
Source: Nucleic Acids Res. 2021 Oct 19;50(D1):D1069–76. doi: 10.1093/nar/gkab925 (PMC8728255; doi:10.1093/nar/gkab925)

PopHumanVar: An Interactive Application for the Functional Characterization and Prioritization of Adaptive Genomic Variants in Humans

Aina Colomer-Vilaplana, Jesús Murga-Moreno, Aleix Canalda-Baltrons, Clara Inserte, Daniel Soto, Marta Coronado-Zamora, Antonio Barbadilla and Sònia Casillas

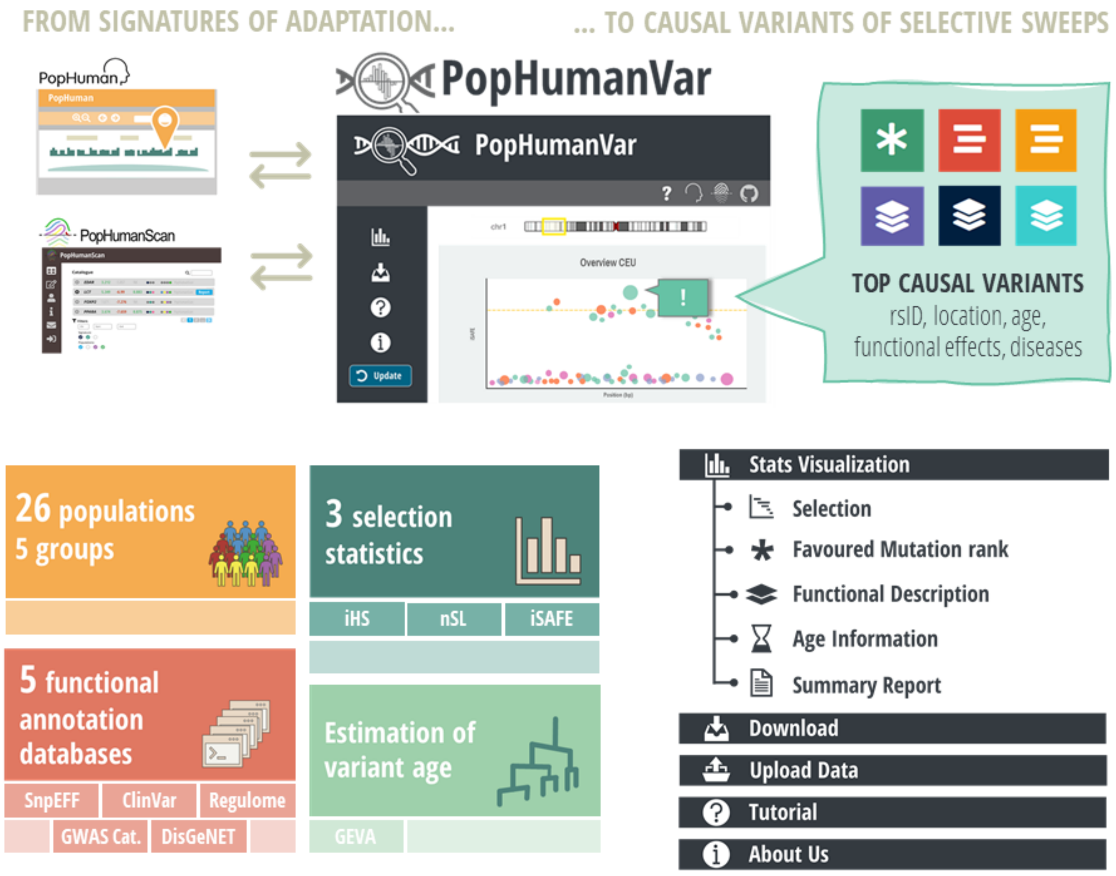

SUPPLEMENTARY DATA

Usage of PopHumanVar on two additional genomic regions which are well-studied ‘gold standard’ examples of selective sweeps in humans: (A) the *ACKR1* (*DARC*) gene region, associated to resistance to *vivax* malaria in Africans; and (B) the *LCT/MCM6* gene region, associated to lactase persistence in Europeans.

Reference: Szpak, M., Xue, Y., Ayub, Q. and Tyler-Smith, C. (2019), How well do we understand the basis of classic selective sweeps in humans?. FEBS Lett, 593: 1431-1448. https://doi.org/10.1002/1873-3468.13447

ACKR1 (*DARC*)

The *Atypical Chemokine Receptor 1* (*ACKR1*) gene on chromosome 1, previously known as *DARC*, the *Duffy Antigen Receptor for Chemokines*, is a glycosylated membrane protein which encodes the Duffy blood group antigen. It is expressed on human red blood cells, endothelial cells, epithelial cells in the lung and kidneys, and cerebellum Purkinje cells. It binds the malarial parasites *Plasmodium vivax* and *Plasmodium knowlesi*, and plays a role in inflammatory responses. The red blood cells homozygous for the null allele (Duffy O blood group) are resistant to parasitic invasion by *P. vivax*. The SNP **rs2814778 (T>C)** occurs in the 5’ untranslated region of the *ACKR1* locus, in a consensus binding site for GATA1, a red blood cell specific transcription factor. The derived C allele is associated with a lower *ACKR1* gene expression and is almost fixed in most African populations. In the GWAS Catalog, it has been significantly associated with several white blood cell traits.

Even though the *ACKR1* gene locus is a well-studied case of adaptation in African populations, the region is not reported in the PopHumanScan catalog. Focusing on one of the East Asian populations (ACB), rs2814778 is the top prioritized variant in PopHumanVar, with the modest iSAFE value of 0.06686.

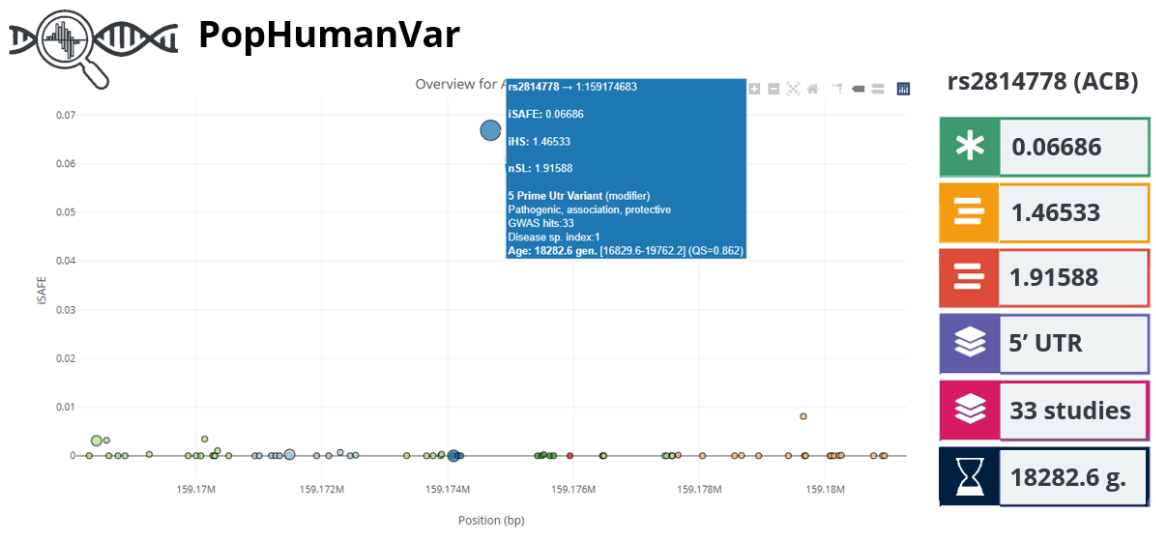

LCT/MCM6

Lactase persistence, the persistence of the expression of *lactase* (*LCT*) phlorizin hydrolase enzyme in intestinal cells in adults, is a heritable, autosomal dominant condition which allows the digestion of the milk sugar lactose during adulthood. The cis-regulatory variant **rs4988235 (G>A)** has been associated with this phenotype in Europeans, and in some West, Central and South Asian populations. It lies ~14 kbp upstream of *LCT* in an intron of *MCM6*, and controls the expression of *LCT*. Other variants within the same intron of *MCM6* have also been associated with lactose tolerance in some African and Arab populations. In the GWAS Catalog, the derived A allele is associated with body mass index, hip circumference and blood protein levels. Alleles in this region associated with the persistence of *LCT* and the ability of adults to digest lactose were selected in several cultures, coupled with the development of dairy farming during the last 10000 years.

The *LCT* gene locus is one of the regions reported in the PopHumanScan catalog. It shows multiple signatures of adaptation, including two statistics based on linkage disequilibrium (iHS and XP-EHH), as well as Fu and Li's D and the McDonald and Kreitman test (MKT), for several human populations all around the world, but mostly European and African populations. Focusing on one of the European populations (CEU), rs4988235 is the top prioritized variant in PopHumanVar, with top ranked values for iSAFE (0.70406) and iHS (4.11907).

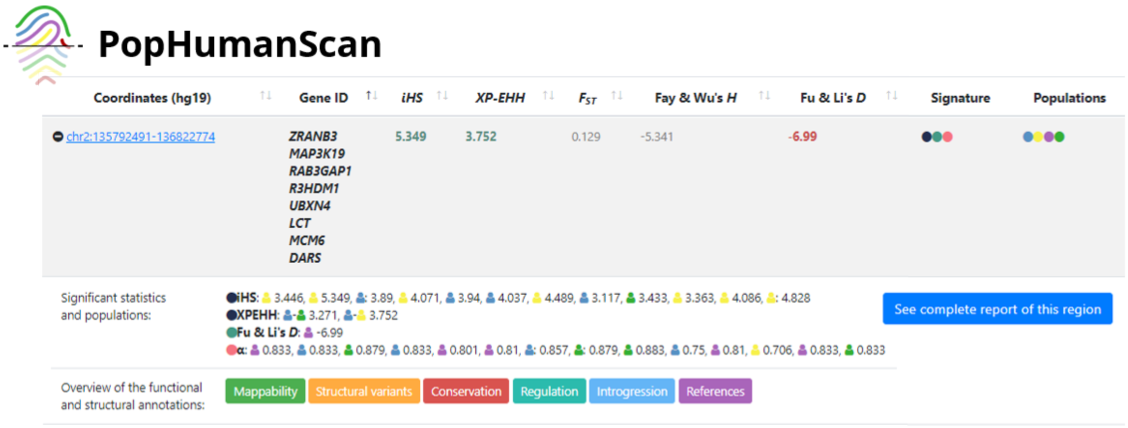

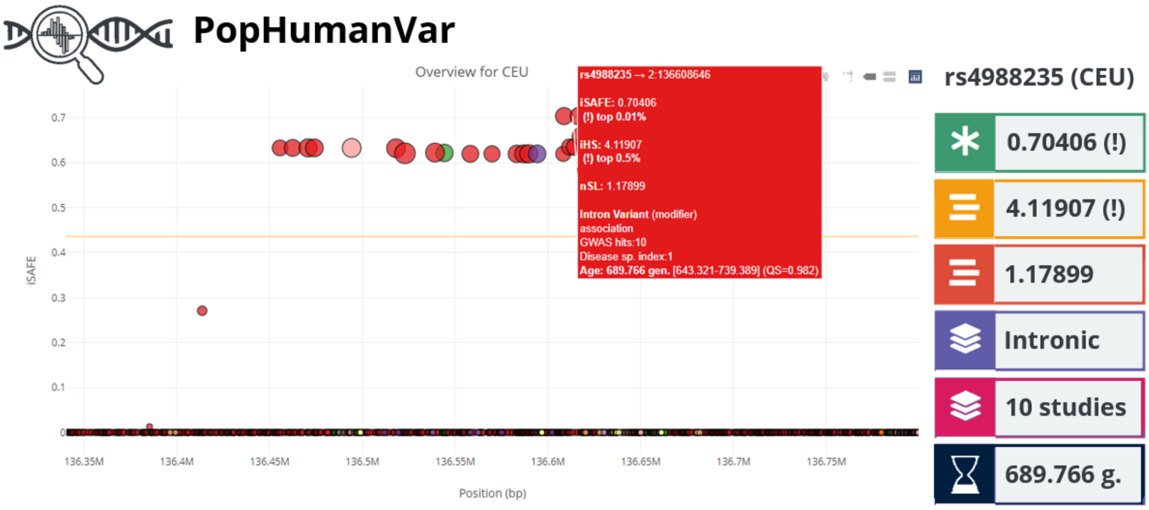

Supplement: gkab925_Supplemental_File [file gkab925_supplemental_file.pdf]
